# Giant mature teratoma in thymic tissue removed with uniportal vats approach

**DOI:** 10.1016/j.ijscr.2019.11.031

**Published:** 2019-11-27

**Authors:** F. Carannante, L. Frasca, V. Marziali, F. Longo, P. Crucitti

**Affiliations:** Department of Thoracic Surgery, Università Campus Bio-Medico, Via Alvaro del Portillo 21, 00128 Rome, Italy

**Keywords:** Thoracic surgery, Uniportal VATS, Teratoma, Thymic tissue

## Abstract

**Introduction:**

Teratomas are tumours composed by different tissues derived from one or more of the three primitive germ cell layers. The frequency of mediastinal teratomas ranged from 1 to 5 %, in most cases with localization in the anterior/superior mediastinum.

**Presentation of case:**

We report a case report of a 29 years old male who presented an occasional and asymptomatic mediastinal mass.

Computer Tomography (CT) scan showed a 6.8 × 4.5 cm mass in the anterior mediastino, located below the left brachio-cephalic vein, next to aortic arch and left pulmonary artery. Complete excision of the mass “en block” with anterior mediastinal adipose tissue was achieved, using uniportal VATS approach. The postoperative course was regular, without air leak or other pulmonary complication.

**Discussion:**

VATS technique is minimally invasive and, moreover, it is characterized by a shorter recovery period, a minor blood loss and a shorter hospital stay. VATS has been advocated since 2010 for pulmonary resections, but today it is also performed for mediastinal intervention and a series of reports have demonstrated that it is feasible and safe.

**Conclusion:**

The report aims at pointing out the possibility of undergoing this kind of surgery not only in case of pulmonary mass (on which the previous literature has focused) but also when mediastinum lesions occur, as in our case.

## Highlights

• Teratomas are tumours composed by different tissues derived from one or more of the three primitive germ cell layers. The frequency of mediastinal teratomas ranged from 1 to 5 %, in most cases with localization in the anterior/superior mediastinum.
• VATS technique is minimally invasive, and it is characterized by a shorter recovery period, a minor blood loss and a shorter hospital stay. VATS has been advocated since 2010 for pulmonary resections, but today it is also performed for mediastinal intervention and a series of reports have demonstrated that it is feasible and safe.
• We demonstrate that uniportal VATS could be used also to remove mediastinal giant mass, without complications for patients, with a reduction hospital stay, less post-operative pain and better cosmetic results.

## 1 Introduction

Teratomas are tumours composed by different tissues derived from one or more of the three primitive germ cell layers [1]. The most common site where teratomas grows is sacro-coccygeal area, followed by ovary, head and neck, retroperitoneum, mediastinum, testes, and central nervous system [2].

The frequency of mediastinal teratomas ranged from 1 to 5 % [3], in most cases with localization in the anterior/superior mediastinum [2,3].

Mediastinal mature teratoma is a rare benign neoplasia, but it could lead to functional problems due to its position. Often the diagnosis could be difficult, and the mass could be adherent to major organs, major vessels as well as the heart, nerves and lungs, causing intra-operative complications [4].

The differential diagnosis of the anterior mediastinal neoplasia is between thymic tumours, thyroid tumours, pericardial cysts, lymphomas.

Uniportal Video Assisted Thoracic Surgery (Uni-VATS) is an emergent method for mediastinal dissection. Previous studies demonstrated the feasibility of the Uni-VATS approach to excise mediastinal teratomas [5]. The hospital stay, chest tube durations, the operative time, duration of postoperative ventilator use, and length of Intensive Care Unit (ICU) stay were shorter in the Uni-VATS group than in the open procedure group [[4], [5], [6]].

Therefore, the presence of symptoms should be considered a relative contraindication of VATS for teratoma resection [4]. The open procedure could be preferred for large mediastinal masses; however, tumour size is not a sufficient factor for determining the surgical method [6]. In particular, our group published a case report of an ectopic thoracic thyroid removed by uniportal VATS approach [7] and Dr. Diego Gonzalez-Rivas removed a tumour of 12.5 cm × 10 cm through a 6-cm wound, with no intra-operative and post-operative complications [5].

Possible complication of this type of surgery are oozing or bleeding from vessels such as the innominate or thymic vein [8].

This work has been reported in line with the SCARE criteria [9].

## 2 Case report

We report a case report of a 29 years old male who presented an occasional and asymptomatic mediastinal mass, discovered during investigations for an orthopaedic disease.

**Fig. 1:**
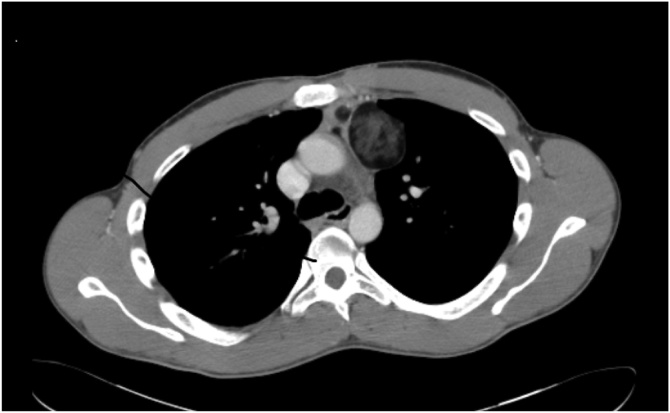
CT scan.

Computer Tomography (CT) scan showed a 6.8 × 4.5 cm mass in the anterior mediastino, located below the left brachio-cephalic vein, next to aortic arch and left pulmonary artery. The neoplasia was poly-lobulated with internal hypodense septa, inhomogeneous content and little capsular calcifications (Fig. 1).

**Fig. 2:**
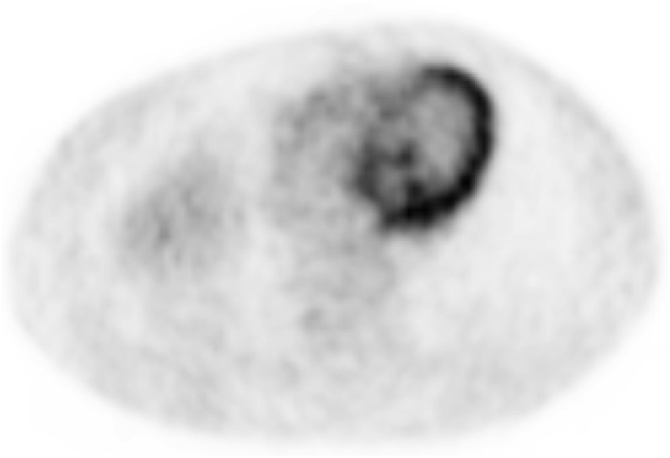
CT/PET scan.

Synchronized CT with 18-fluorodeoxyglucose-positron emission tomography (18FDG-PET/CT) scanning showed a focal hyper-uptake at the anterior-inferior part of the mass with Maximum Standardized Uptake value (SUVmax) of 5.9. A small area behind the sternum body showed a weak hyper-uptake. The remaining part of the mass didn't show an increase glycolic activity (Fig. 2).

The patient was positioned in right lateral decubitus position. Uni-VATS was performed through a left lateral thoracotomy of 3 cm at 4th intercostal space between middle and anterior axillary line. The surgical procedure was performed under general anaesthesia. The patient was intubated with double lumen endotracheal tube to allow a selective ventilation of the right lung. This kind of intubation is used for airway management and also to separate the left lung from the surgical field.

The mass appeared have infiltrate the mediastinal pleura and left phrenic nerve. The latter couldn't be dissociated by the mass so it was dissected above and below the mass.

Complete excision of the mass “en block” with anterior mediastinal adipose tissue was achieved.

We didn't use CO2. A 24 fr chest tube was placed in the pleural space. The operative time was about 90 min without relevant blood loss.

The macroscopic analyse described 8.5 × 5 × 3 cm mass in which could be found solid yellow-white areas, cystic milky areas and hair.

Histology revealed a mature teratoma without atypical cells. The surrounding tissues presented multilocular thymic cyst and chronic granulomatous inflammations with giant cells.

**Fig. 3:**
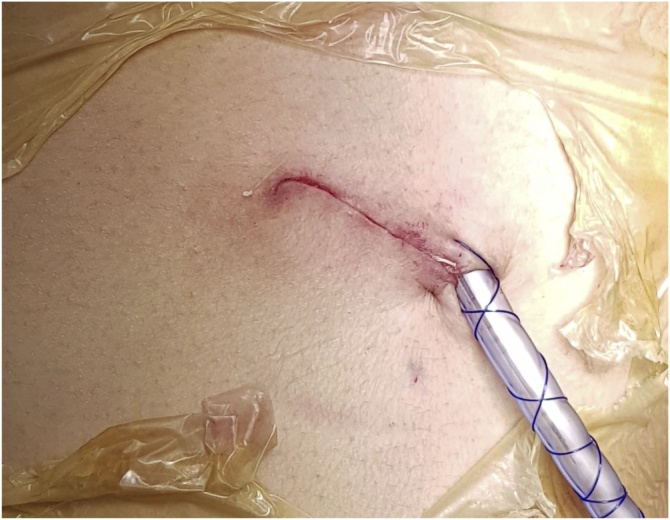
Surgical incision with pleural drainage.

The postoperative course was regular, without air leak or other pulmonary complication. The Chest tube has been removed in the second postoperative day (Fig. 3).

The hospital stay lasted 2 days. Patient had dysphonia and weak voice, cough for liquids. After discharge the patient has been prescribed a phoniatric visit, that found left vocal cords paralysis in paramedian position and left arytenoid asymmetry.

We visited the patient 10 and 30 days after discharge to check the wound and the patient’s general condition. The RX imagine showed no complications. Speech therapy rehabilitation was prescribed, and his condition improved in a month. The voice got better, and the mobility of the vocal cord returned almost normal.

## 3 Discussion

Uniportal Video Assisted Thoracic Surgery was recently stand out as a feasible and safe technique.

This surgical approach is routinely used to perform various thoracic interventions, such as pulmonary lobectomy and pulmonary atypical resection.

For anterior mediastinal tumor resection, various surgical methods are available, including sternotomy, anterior lateral thoracotomy, and VATS. Thus, the development of mini-invasive surgery has gradually changed the surgical technique.

In conclusion, we demonstrate that uniportal VATS could be used also to remove mediastinal giant mass, without complications for patients, with a reduction hospital stay, less post-operative pain and better cosmetic results. We believe that in the hands of appropriately experienced surgeons, this type of surgical approach of mediastinal could be a safe, effective option even for such kind of patient.

## Sources of funding

The Authors disclose no sources of funding for research.

## Ethical approval

This is a case report. It’s exempt from ethical approval.

## Consent

Written informed consent was obtained from the patient for publication of this case report and accompanying images. A copy of the written consent is available for review by the Editor-in-Chief of this journal on request

## Author contribution

F. Carannante: study design, data collections, data analysis, and writing.

L. Frasca: data collections, data analysis

V. Marziali: study design, data collections, data analysis, and writing.

F. Longo: study design

P. Crucitti: reviewer.

## Registration of research studies

This study is not a first in man study and the registration in a publicly accessible database is not required.

## Guarantor

Dr. P. Crucitti.

## Provenance and peer review

Not commissioned, externally peer-reviewed.

## Declaration of Competing Interest

The Authors disclose no conflicts
